# Natural Killer Cells in Graft-versus-Host-Disease after Allogeneic Hematopoietic Cell Transplantation

**DOI:** 10.3389/fimmu.2017.00465

**Published:** 2017-04-25

**Authors:** Federico Simonetta, Maite Alvarez, Robert S. Negrin

**Affiliations:** ^1^Division of Blood and Marrow Transplantation, Department of Medicine, Stanford University School of Medicine, Stanford, CA, USA; ^2^Division of Hematology, Department of Oncology, Geneva University Hospitals, University of Geneva, Geneva, Switzerland

**Keywords:** natural killer cells, graft-versus-host-disease, bone marrow transplantation, HSCT, tolerance

## Abstract

Allogeneic hematopoietic cell transplantation (HCT) is a well-established therapeutic modality effective for a variety of hematological malignancies but, unfortunately, is associated with significant morbidity and mortality related to cancer relapse as well as to transplant-related complications including graft-versus-host-disease (GvHD). Natural killer (NK) cells are the first donor-derived lymphocyte subset to recover after HCT, and their crucial role in protection against cancer relapse and infections is well established. Conversely, the role played by NK cells in GvHD is still controversial. Early studies suggested a participation of NK cells in GvHD induction or exacerbation. Subsequently, experimental evidence obtained in mice as well observational studies performed in humans led to a model in which NK cells play a regulatory role in GvHD by repressing alloreactive T cell responses. This widely accepted model has been recently challenged by clinical evidence indicating that NK cells can in some cases promote GvHD. In this review, we summarize available knowledge about the role of NK cells in GVHD pathogenesis. We review studies uncovering cellular mechanisms through which NK cells interact with other immune cell subsets during GvHD leading to a model in which NK cells naturally suppress GvHD through their cytotoxic ability to inhibit T cell activation unless exogenous hyperactivation lead them to produce proinflammatory cytokines that can conversely sustain T cell-mediated GvHD induction.

## Introduction

Natural killer (NK) cells are the first donor-derived lymphocyte subsets to recover after hematopoietic cell transplantation (HCT), preceding by several months the reconstitution of adaptive T and B lymphocytes. NK cells have been the focus of significant attention in the HCT field over the last four decades. Studies of the role of NK cells in bone marrow engraftment demonstrated that host NK cells persisting after conditioning can contribute to graft rejection (1) while donor NK cells can promote hematopoietic engraftment (2). At the same time, several preclinical and clinical studies focusing on NK cell alloreactivity in anticancer responses identified donor NK cells as crucial players in preventing cancer relapse after HCT for hematologic malignancies (3, 4). Less well established, however, is the role of NK cells in graft-versus-host-disease (GvHD), a major complication of HCT. While the classical model of GvHD pathogenesis includes, together with donor-derived T cells, donor-derived NK cells in the immune-pathological activation leading to GvHD (5), evidence from preclinical models as well as from studies in human HCT recipients led to a more complex picture where NK cells could either promote or prevent GvHD.

In this review, we summarize the available knowledge about the role of NK cells in GvHD pathogenesis. After reviewing preclinical and clinical studies uncovering cellular mechanisms through which NK cells interact with other immune cell subsets during GvHD, we propose a new model in which distinct effector mechanisms determine the pathogenic or regulatory role of NK cells in promotion or control of GvHD, respectively. Finally, we discuss the impact that GvHD can in turn have on NK cell biology and the potential consequences in the context of HCT.

## Early Studies

The first study suggesting a relationship between NK cells and GvHD development was reported by Lopez and coworkers from the Sloan Kettering Cancer Center showing a significant association between GvHD development and pre-transplant levels of NK cell activity, as measured by cytotoxic assays performed using herpes simplex virus type 1-infected fibroblast as target cells, in peripheral blood of a small and heterogeneous cohort of 13 patients undergoing different protocols of HCT (6). Importantly, most of the patients included in the series underwent HCT after myeloablative conditioning, and no information was provided about NK cell activity after transplantation. Shortly thereafter, Livnat et al. (7) and Dokhelar et al. (8) addressed the same issue assessing NK cell activity against the K562 leukemic cell line both before and after HCT and obtained contradictory results finding either no relationship (7) or a positive association (8) between early posttransplant NK cell activity and GvHD development. Despite the contradictory conclusions obtained and the limitations of the studies including the heterogeneity of the patients cohorts as well as of the analytical methods employed, these early studies opened the way to numerous studies addressing the role of NK cells in GvHD.

A first approach has been to investigate the presence of NK cells in GvHD target organs. In the mouse parent-into-F1 (P > F1) model of GvHD, increased NK cell activity measured against YAC lymphoma target cells was detected in spleen (9–11), lymph nodes (9, 10), thymus (9, 12), and intestinal intraepithelial lymphocytes (10) from mice with active GvHD. Similarly, in murine minor mismatch HCT models, large granular lymphocytes displaying an immunophenotype characteristic of NK cells infiltrated the skin (13), liver, and intestine (14) from animals with acute GVHD. Importantly, the use of congenic markers demonstrated that these cells were of donor origin (14). Accordingly, the study of biopsies obtained from skin (15–17), liver (18, 19), and intestinal (20) of patients with acute GvHD showed the presence of NK cells among the lymphoid population infiltrating these GvHD target tissues. The study of biopsies obtained from female patients transplanted with male donor grafts confirmed in humans the donor origin of the NK cells infiltrating tissues during GvHD (16). The target tissues infiltration by NK cells during GvHD, both in mice and humans, supported a model in which NK cells may induce, or at least contribute to, GvHD development. Attempts were, therefore, made to obtain experimental evidence supporting this hypothesis, first by using NK cell depleting antibodies directed against the cell surface glycolipid asialo GM1 or to the cell surface NKR-P1 family receptor NK1.1. However, results from reports employing this approach were inconsistent, few studies suggested a reduction of GvHD upon treatment of recipients (21–23) while most studies employing antibody depletion on donor cells showed only minimal if any impact on GvHD development (23–27). This discrepancy suggested that depleting antibodies exerted their effect through the depletion of an effector cell population appearing after HCT rather then by depleting NK cells contained in the graft. Further, the epitopes recognized by anti-asialo GM1 and anti-NK1.1 antibodies are expressed by several immune cell subsets other than NK cells, including activated T cells involved in GvHD development (28–30), making it impossible to distinguish between an NK and a T cell directed effect. Ghayur et al. used a complimentary approach employing beige mice carrying a homozygous *bg* mutation that leads to severe deficiency in NK cell function. Adoptive transfer of *bg/bg* splenocytes failed to induce GvHD in a P > F1 model, while transfer of heterozygous +/*bg* induced hepatic GvHD, suggesting that donor NK cells were responsible for GvHD induction (31). However, even in this model, a functional deficit in adaptive T cells from beige mice complicates the interpretation of the results (32, 33).

## NK Cell Cytotoxic Functions and GvHD Prevention

While murine models based on antibody depletion or genetic alteration of NK cells failed to provide consistent evidence for a role of NK cells in GvHD pathogenesis, the adoptive transfer of NK cells offered unexpected insights. In an attempt to promote bone marrow engraftment in a major mismatch murine model, Murphy and coworkers adoptively transferred NK cells purified from C.B-17 severe combined immunodeficiency (SCID) (H-2^d^) mice into lethally irradiated C57BL/6J (H-2^b^) mice together with non-T-cell depleted bone marrow cells from BALB/cJ (H-2^d^) mice with or without splenocytes (2). In mice not receiving splenocytes, transferred NK cells did not induce GvHD, thus questioning the NK GvHD-inducing potential suggested by antibody depletion studies. More interestingly, in mice receiving splenocytes, activated NK cells prevented the development of GvHD that invariably lead to death of mice injected with BM cells and splenocytes alone. This unexpected result revealed not only that NK cells can be adoptively transferred safely in this major mismatch model without inducing GvHD but also that they can prevent T cell-mediated GvHD development. The results of this first study were confirmed during the years by several other reports (3, 34–39) and numerous studies in humans suggested that higher numbers of NK cells (40–47) and the presence of NK cell alloreactivity (3, 4, 48–50) reduce GvHD development.

In particular, NK cell alloreactivity has been found to be crucial for NK cell-mediated protection from GvHD. Ruggeri et al. showed in a major mismatch HCT murine model that alloreactive Ly49 ligand-mismatched NK cell infusion prevented T cell-induced GvHD, while administration of even large numbers of non-alloreactive Ly49 ligand-matched NK cells provided no protection (3). These results were subsequently confirmed by Lundqvist et al. who further extended this observation showing that, although inefficient in preventing GvHD, Ly49 ligand-matched NK cells displayed an antitumor activity similar to Ly49 ligand-mismatched NK cells (35). The need of Ly49 ligand-mismatch for GvHD control by NK cells prompted some investigators to silence Ly49C to induce alloreactivity with promising results (51). Alloreactive NK cells were shown to indirectly inhibit T cell proliferation and GvHD induction by depleting antigen-presenting cells (APCs) (3, 38) through their cytolytic activity, the c-Kit^−^CD27^−^CD11b^+^ NK cells being the most potent in this effect (38). In particular, the expression of the activating receptor KIR2DS1, which binds to HLA-C2, seems to contribute to the APCs’ killing and it was even able to override the inhibition mediated by the expression of the inhibitory receptor NKG2A, which binds to HLA-E in humans or Qa-1b in mouse (50). Similarly, proportions of donor-derived NK cells expressing the activating receptor CD94/NKG2C, which recognize as well HLA-E/Qa-1b, were lower in HLA-matched and HLA-mismatched HCT recipients with acute or chronic GvHD compared with patients without GvHD (52). Accordingly, patients with acute or chronic GvHD displayed a lower ratio of CD94/NKG2C to CD94/NKG2A on NK cells suggesting a competition for the same ligands between NKG2C and NKG2A that would result in NK cell activation or suppression, respectively (52). Finally, Ghadially et al. suggested that NK cell-mediated killing of APC during GvHD is mediated by the stimulation of NKp46 receptor by still unknown ligand(s) expressed by dendritic cells (DCs) as the absence of NKp46 on donor NK cells leads to increased stimulation of donor T cells by DCs (53), resulting in increased tissue damage (54).

**Figure 1:**
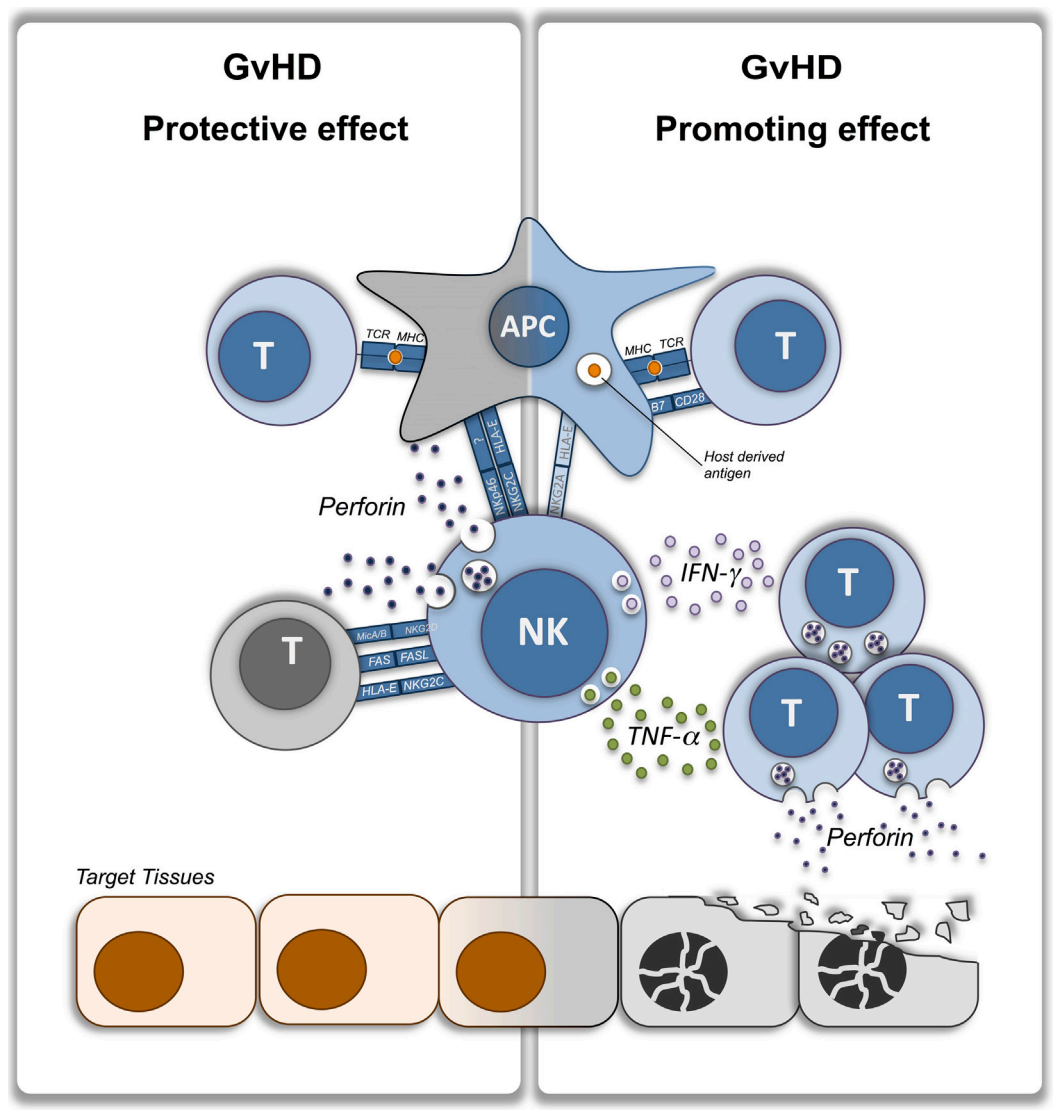
**NK cells contribution to graft-versus-host-disease (GvHD) protection or induction**. Immunological interactions leading to GvHD protective (left panel) or promoting (right panel) effect of NK cells. Donor immune cells are depicted in blue while host target cells are depicted in orange. NK, natural killer; T, T lymphocyte; APC, antigen-presenting cell; IFN-γ, interferon-γ; TNF-α, tumor necrosis factor-α.

In addition to this indirect, APC-killing mechanism, others and we have shown that NK cells can suppress GvHD by directly lysing activated T cells. *In vitro* evidence obtained in murine (55) and human (56, 57) cells showed that T cells during activation upregulate stress molecules acting as ligands for the NK activating receptor NKG2D, thus becoming targets of NK cell-mediated killing. In a major mismatch HCT model, we showed that allogeneic T cells upregulate the NKG2D ligand Rae1γ and perhaps other molecules during GvHD and thus become susceptible to NK cell-mediated killing through a NKG2D-dependent cell lysis (37). Noval Rivas and coworkers obtained very similar results in a minor mismatch model of chronic GvHD induced by adoptive transfer of monoclonal anti-male CD4 T cells into lymphopenic male mice (58). Interestingly, we observed in our system an increased ratio of splenic donor regulatory T cells (Treg) to total donor conventional CD4^+^ and CD8^+^ T cells (Tcon) in the presence of NK cells, suggesting a differential susceptibility of Treg and Tcon to NK cell-mediated cell lysis leading to an immune-regulatory environment that eventually contributes to GvHD suppression (37). Direct T cell killing by NK cells can, therefore, be considered as a complimentary mechanism of GvHD suppression, in addition to the aforementioned modulation by APC-killing, which can be particularly important at GvHD tissues sites. Accordingly, we have shown that, after transplantation, NK cells traffic to GvHD target organs following a spatial and temporal distribution very similar to T cells (59) offering them the opportunity to target activated T cell at the effector site. However, in contrast to T cells, NK cells have a more limited persistence, which may in part explain their reduced capacity for GVHD induction. Interestingly, GvHD prevention by T cell killing at tissue sites can be exerted as well by residual tissue resident recipient NK cells eventually persisting after conditioning depending on conditioning intensity as it has been recently shown in a minor mismatch murine model (60). T cell killing by NK cells appears to be dependent on both perforin production (37, 60) and FAS-mediated induction of apoptosis (37, 58, 61). Collectively, these models demonstrated that NK cells can suppress GvHD development through their cytotoxic function either directly, by depleting activated alloreactive T cells, or indirectly, by depleting APC and preventing T cell stimulation (Figure 1, left panel).

## NK Cell Cytokine Production and GvHD Induction

In addition to their cytolytic potential, NK cell can modulate immune responses through cytokine production. Whether this mechanism can participate in GvHD prevention by NK cells is unclear. One of the early studies showed that administration of anti-TGFβ monoclonal antibody significantly limited the NK cell suppressive effect on GvHD (34). However, no evidence was provided that NK cells were indeed the source of TGFβ and administration of exogenous TGFβ failed to prevent GvHD development, indicating that TGFβ contribution to GvHD suppression is only partial and through a mechanism still to be completely uncovered.

Although it is unclear if NK cells production of immune-suppressive cytokines can prevent GvHD, it is established that pro-inflammatory cytokine production by NK cells can contribute to GvHD development. In a xenogeneic model, Xun et al. showed that *in vitro* interleukin-2 (IL-2)-activated human NK cells producing interferon-γ (IFN-γ) and tumor necrosis factor-α (TNF-α) were able to induce acute GvHD upon transfer into SCID mice (62, 63). Interestingly, NK cells were found in GvHD target tissues in juxtaposition to damaged cells and produced *in situ* IFN-γ and TNF-α (62). Although the limitations of the xenogeneic model should be taken into account, the results from the aforementioned experiments suggest that, when pre-activated to produce the pro-inflammatory cytokines IFN-γ and TNF-α, NK cells can indeed promote rather than prevent GvHD development. In accordance, while NK depletion by NK1.1 antibodies had no effect on GvHD when employed on steady-state donor splenocytes (25), it significantly prevented GvHD when employed on splenocytes obtained from donor mice previously treated with the toll-like receptor 3 stimulator polyinosinic:polycytidylic acid (poly I:C) (64, 65) by reducing IFN-γ production (65). Further, higher proportions of IFN-γ-producing NK cells after HCT have been shown to be associated in humans with an increased incidence of acute GvHD (66). Collectively, these studies provide evidence for a promoting role of NK cells in GvHD, opposite from the suppressive role exerted by cytolysis, through the production of pro-inflammatory cytokines that may act directly to induce cell damage or indirectly by increasing T cell-mediated tissue damage through their well-known property to increase MHC expression (Figure 1, right panel). This model can be useful in the interpretation of the otherwise surprising results recently reported by Shah and coworkers (67). Most studies involving adoptive transfer of NK cells into HCT recipients failed to observe GvHD induction after infusion (68–70) (Table 1). Similarly, studies assessing the potential of adoptively transferred allogeneic haploidentical NK cells into lymphodepleted patients in non-allogeneic HCT settings did not observe any cases of acute GvHD (71–75) (Table 1). Few studies reporting the development of acute GvHD after allogeneic NK cell adoptive transfer (76–78) were unable to establish a causative relationship between the NK cell infusion and GvHD development because of other potential contributing factors including immune-suppression discontinuation (76) or residual T cell contamination of the administered cell product (77). Conversely, the report by Shah and coworkers provide some evidence for an NK cell involvement in GvHD development. The authors reported the development of GvHD in five out of nine recipients of HLA-matched, T-cell-depleted peripheral blood HCT upon adoptive transfer of donor-derived IL-15/4-1BBL-activated NK cells (67). The direct involvement of donor NK cells in GvHD was suggested by their presence in the lymphoid infiltrate found in biopsies of GvHD involved tissues (67). However, despite the fact that grafts contained very low numbers of T cells as a result of T cell depletion by CD34 positive selection, several issues suggested that the NK-cell-promoting role on GvHD could have been mediated by an indirect effect on T cells. First, a higher proportion of patients developing GvHD received grafts from unrelated donors, therefore, were provided with a higher alloreactive potential, compared to patients not developing GvHD (67). Second, patients developing GvHD displayed more rapid T-cell engraftment, as revealed by day 14 and day 28 CD3-chimerism, compared with patients not developing GvHD (67). Moreover, it should be noted that patients were free of T-cell directed immune-suppressive treatment at the time of adoptive transfer. Importantly, the timing of the administration of the NK cells could have been another factor pushing the balance toward GvHD induction. Patients from the aforementioned report (67) received the pre-activated NK cells around the time of engraftment. Murine studies have demonstrated the importance of the timing of NK cell administration on GvHD prevention, showing no benefit of delayed treatment (37) and even a potential for GvHD exacerbation when NK cells were administered at later time points (34), although in these latter experiments IL-2 was administered at the same time as the NK cells and could have contributed to the phenomenon. This opposing effect can be related with the production of IFN-γ that has been shown to inhibit GVHD when provided early after HCT and to exacerbate GVHD when acting at a later time (79). Considering all of these factors, it can be speculated that the administration of highly pre-activated NK cells can enhance clinically undetectable T-cell alloreactivity through the production of pro-inflammatory cytokines (Figure 1, right panel) and that this functional aspect can, therefore, prevail on their GvHD-protective cytotoxic activity (Figure 1, left panel), thus promoting GvHD development.

**Table 1:** **Acute graft-versus-host-disease (GvHD) development reported in published natural killer (NK) cells adoptive transfer clinical trials**.

Reference	*N*	Age	Disease	Donor type	Conditioning	Time from allo-hematopoietic cell transplantation (HCT)	Cell isolation	NK cells preparation	Cell dose (10^6^/kg)	Combined therapy	Acute GvHD
Passweg et al. (68)	5	Adult	AML	Haploidentical	Etoposide	Post allo-HCT (day +3 to +26)	CD3 depletion	Fresh	6.9–14.1	–	0/5 (0%)
			CML		Cy/TBI/ATG		CD56 selection				
Miller et al. (71)	19	Adult	AML	Haploidentical	Cy/Flu	No allo-HCT	CD3 depletion	Interleukin-2 (IL-2) activated	0.1–20	IL-2	0/43 (0%)
			Solid tumors								
Rubnitz et al. (72)	10	Ped	AML	Haploidentical	Cy/Flu	No allo-HCT	CD3 depletion	Fresh	5–8	IL-2	0/10 (0%)
							CD56 selection				
Yoon et al. (76)	14	Adult	AML	Haploidentical	Bu/Flu/ATG	Post allo-HCT (day +43 to +50)	CD34 selection	*In vitro* differentiated	N/A	–	1/14 (7%) (1 grade II)
			MDS	HLA-mismatched			*In vitro* differentiation				
			ALL								
Curti et al. (73)	13	Adult	AML	Haploidentical	Cy/Flu	No allo-HCT	CD3 depletion	Fresh	1.11–5	IL-2	0/13 (0%)
							CD56 selection				
Stern et al. (77)	16	Adult	AML	Haploidentical	MAC/ATG or OKT3	Post allo-HCT (day +3 to +40)	CD3 depletion	Fresh	8–76	–	4/16 (25%) (1 grade II, 2 grade III, 1 grade IV)
		Ped	ALL				CD56 selection	Cryopreserved			
			Solid tumors								
Klingemann et al. (74)	13	Adult	HL	Haploidentical	None	No allo-HCT	CD3 depletion	IL-2 activated	0.1–20	–	0/13 (0%)
			NHL								
			MM								
Bachanova et al. (75)	57	Adult Ped	AML	Haploidentical	Cy/Flu	No allo-HCT (*n* = 53) Post allo-HCT (*n* = 4)	CD3 depletion	IL-2 activated	3.4–15	IL-2	0/57 (0%)
							±CD19 depletion			IL2DT	
							±CD56 selection				
Choi et al. (69)	41	Adult	AML/MDS	Haploidentical	Bu/Flu/ATG	Post allo-HCT (day +14 to +21)	CD3 depletion	*Ex vivo* expanded	20–500	–	9/41 (21%) (2 grade I, 2 grade II, 5 grade III–IV)
			ALL								
			Lymphoma								
Shah et al. (67)	9	Adult	Sarcomas	HLA-matched sibling/unrelated donor	Cy/Flu/Melph	Post allo-HCT (day +7 to +35)	CD3 depletion	IL-15/4-1BBL activated	0.1–1	–	5/9 (55%) (1 grade II, 3 grade IV, 1 non-gradable)
		Ped					CD56 selection				
Lee et al. (78)	21	Adult Ped	AML	Haploidentical	Bu/Flu	Post allo-HCT (day −8)	CD3 depletion	IL-2 activated	0.02–8.32	IL-2	7/21 (33%) (5 grade II, 2 grade III)
			MDS				CD56 selection				
			CML								
Jaiswal et al. (70)	10	Adult	AML	Haploidentical	Treo/Flu/TBI	Post allo-HCT (day +7)	CD56 selection	Fresh	1.7–17.7	–	0/10 (0%)
		Ped	CML		PTCy						

*ALL, acute lymphoblastic leukemia; AML, acute myeloid leukemia; ATG, antithymocyte globulin; BM, bone marrow; Bu, busulfan; CML, chronic myeloid leukemia; Cy, cyclophosphamide; Flu, fludarabine; HL, Hodgkin’s lymphoma; MDS, myelodysplastic syndrome; Melph, melphalan; NHL, non-Hodgkin lymphoma; MAC, myeloablative conditioning; MM, multiple myeloma; PTCy, posttransplant cyclophosphamide; TBI, total body irradiation; Treo, treosulfan*.

## GvHD Modulation of NK Cells

While NK cells may positively or negatively participate in GvHD development, the GvHD process can in turn affect NK cell biology. Pattengale and coworkers were the first to demonstrate in murine models that acute but not chronic GvHD induce a marked decrease in NK cell activity associated with an impaired production of IFN-γ (80). NK cell reconstitution appears to be significantly delayed by acute GvHD in mice (81) and by acute and chronic GvHD in humans (42, 82–85). Recent evidence from a murine model of GvHD suggest that activated T cells could limit NK cell access to IL-15 through direct competition for this cytokine necessary for NK cell development and homeostasis, administration of exogenous IL-15 being able to restore NK cell reconstitution (81). In addition to its quantitative effect, GvHD induces qualitative defects on NK cells ultimately leading to impaired function. Bunting and coworkers recently showed in mice that, during GvHD, donor NK cells display a hyperactivated phenotype associated with increased signs of apoptosis and autophagy (81). Importantly, they showed that GVHD-induced alterations in NK cells resulted in defective *in vivo* cytotoxicity resulting in a reduction of graft-versus-leukemia effect and an impaired control of cytomegalovirus infection (81). This dysfunctional status induced by GvHD is reminiscent of the NK cell exhaustion phenomenon we observed upon chronic proliferation, characterized by an impaired transcriptional machinery as revealed by the downregulation of the Tbox transcription factors Eomesodermin and Tbet (86). Accordingly, we reported in humans that exhaustion is increased in NK cells after HCT and is further exacerbated in NK cells from patients with acute GvHD (87).

## Concluding Remarks

Despite major efforts undertaken during many years to better understand NK cells biology in the context of HCT, the role of NK cells during GvHD remained elusive because of conflicting evidence coming from different experimental approaches. NK cells are capable of both effector and regulatory functions. This pleiotropic nature of NK cells is likely responsible for the variable and even conflicting roles that NK cells can play during GvHD. We hope our model (Figure 1) will help interpret this apparent contradiction. Importantly, clarifying the impact of NK cell activation status on their GvHD induction potential will hopefully contribute to the optimization of cell manufacturing procedures to maximize allogeneic NK cell antitumor potential while preventing GvHD induction.

## Author Contributions

FS wrote the manuscript and designed the figure. MA critically revised the work for important intellectual content and edited the manuscript. RN edited the manuscript and provided overall guidance.

## Conflict of Interest Statement

The authors declare that the research was conducted in the absence of any commercial or financial relationships that could be construed as a potential conflict of interest.
